# Schrödinger's Red Beyond 65,000 Pixel‐Per‐Inch by Multipolar Interaction in Freeform Meta‐Atom through Efficient Neural Optimizer

**DOI:** 10.1002/advs.202303929

**Published:** 2023-12-13

**Authors:** Ronghui Lin, Vytautas Valuckas, Thi Thu Ha Do, Arash Nemati, Arseniy I. Kuznetsov, Jinghua Teng, Son Tung Ha

**Affiliations:** ^1^ Agency for Science, Technology and Research (A*STAR) Institute of Materials Research and Engineering (IMRE) 2 Fusionopolis Way, Innovis #08‐03 Singapore 138634 Republic of Singapore

**Keywords:** machine learning, metasurfaces, monte Carlo tree search, multipole interference, structural colors

## Abstract

Freeform nanostructures have the potential to support complex resonances and their interactions, which are crucial for achieving desired spectral responses. However, the design optimization of such structures is nontrivial and computationally intensive. Furthermore, the current “black box” design approaches for freeform nanostructures often neglect the underlying physics. Here, a hybrid data‐efficient neural optimizer for resonant nanostructures by combining a reinforcement learning algorithm and Powell's local optimization technique is presented. As a case study, silicon nanostructures with a highly‐saturated red color are designed and experimentally demonstrated. Specifically, color coordinates of (0.677, 0.304) in the International Commission on Illumination (CIE) chromaticity diagram – close to the ideal Schrödinger's red, with polarization independence, high reflectance (>85%), and a large viewing angle (i.e., up to ± 25°) is achieved. The remarkable performance is attributed to underlying generalized multipolar interferences within each nanostructure rather than the collective array effects. Based on that, pixel size down to ≈400 nm, corresponding to a printing resolution of 65000 pixels per inch is demonstrated. Moreover, the proposed design model requires only ≈300 iterations to effectively search a thirteen‐dimensional (13D) design space – an order of magnitude more efficient than the previously reported approaches. The work significantly extends the free‐form optical design toolbox for high‐performance flat‐optical components and metadevices.

## Graphical abstract

Using an efficient neural optimizer, the generalized multipolar interference is uncovered in complex photonic structures and realize red structural color with higher color saturation and spatial resolution than any previous reports. The design also exceeds the cadmium red pigments in turns of color saturation.

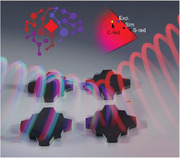

## 1 Introduction

Recent advances in metaoptics have brought many breakthroughs in a wide range of applications, such as optical computing,^[^ ^1^, ^2^, ^3^ ^]^ signal processing,^[^ ^4^, ^5^ ^]^ imaging,^[^ ^6^, ^7^, ^8^ ^]^ and communication.^[^ ^9^, ^10^, ^11^ ^]^ In particular, freeform structures^[^ ^12^, ^13^ ^]^ realized by the inverse design methods enable versatile control of the wave propagation for multifunctional,^[^ ^14^, ^15^, ^16^ ^]^ broadband,^[^ ^17^, ^18^ ^]^ and polarization‐controllable photonic devices.^[^ ^19^ ^]^ However, designing such freeform metadevices often involves optimization in high‐dimensional design space. With increase in dimensionality, available data becomes sparse. Concepts such as proximity, distance, or nearest neighbors are no longer meaningful descriptions of spatial relationships between data points.^[^ ^20^ ^]^ These problems, summed up as “the curse of dimensionality”,^[^ ^21^, ^22^ ^]^ remain an open challenge despite recent impressive results in photonics inverse design,^[^ ^13^, ^22^ ^]^ where there are two main categories of approaches.

The first category involves gradient‐based methods and heuristic algorithms such as Particle Swarm Optimization (PSO), Simulated Annealing (SA), and Differential Evolution (DE).^[^ ^12^, ^13^ ^]^ These algorithms take inspiration from a physical scenario to improve the target function iteratively.^[^ ^23^ ^]^ They usually take a long time to converge, especially for structures with large degrees of freedom (DOF). The adjoint sensitivity analysis method can obtain the gradient of the target function for each point in the design space in just two runs.^[^ ^13^, ^24^ ^]^ However, it is a local optimization process with no guarantee of global extrema. The difficulty in adopting nonquadratic merit functions^[^ ^13^ ^]^ further restricts this algorithm.

The second category consists of data‐driven algorithms that rely on the statistical characteristics of the entire training data, allowing for a more comprehensive overview of the optimization problem. However, the bottleneck is to obtain sufficient high‐quality data. Although classical universal approximation theorems^[^ ^25^, ^26^ ^]^ state that a deep neural network (DNN) is capable of approximating any function and its derivatives with arbitrary accuracy, in practical scenarios, the fine structures of the target function are often lost due to the poor quality or deficiency of the training data and the interpolation error.^[^ ^27^ ^]^ This results in mode collapse,^[^ ^28^ ^]^ where the algorithm can output only a limited number of similar designs and trivial optical responses. In addition, statistical methods are unsuitable for the exploration of underlying physics and devices with exceptional performance since these designs are often found at the anomaly and out‐of‐distribution data points. Given the vast number of training data needed, data‐driven methods are more suitable for similar design tasks with little variations of the design targets.^[^ ^27^ ^]^ The recent approaches combining machine learning with optimization techniques ^[^ ^23^, ^29^, ^30^, ^31^ ^]^ typically use machine learning to provide rough value for further optimization and still suffer from the restrictions of the training data.

The adoption of reinforcement learning (RL)^[^ ^32^ ^]^ for inverse design marks a complete shift of paradigm. Instead of obtaining a direct mapping between the input and the output spaces, the RL technique is a process‐based technique that strives to maximize a reward function iteratively in reaction to the environment. In this regard, it resembles the conventional iterative algorithms. However, the updating rules are learned by the algorithm through continuous trial and error, resulting in a long training time.^[^ ^12^, ^22^ ^]^ Despite a vast interest in both smart machine learning and conventional optimization algorithms,^[^ ^13^, ^22^, ^23^, ^27^, ^33^, ^34^, ^35^, ^36^ ^]^ a global algorithm that combines speed, efficiency, accuracy, diversity of design geometries, and diversity of optical response, which can explore the extrema of target space is still lacking.

In this work, we propose an efficient neural optimizer for resonant nanostructures to achieve desired optical properties with exceptional performance. The freeform geometrical shapes are represented by a low dimensional latent vector, which effectively reduces the dimensionality of the search space. Furthermore, the use of a self‐improving global search algorithm combined with a local search algorithm leads to faster convergence of the optimizing parameters. As a case study, we used this algorithm to tackle an interesting phenomenon: the absence of angle‐independent, high‐saturation red structural color in the natural world.^[^ ^37^, ^38^ ^]^ This phenomenon was found to be related to a universal property of waves that higher‐order resonance modes always accompany their fundamental modes, resulting in reflection peaks in the blue/green regions, hence lower red saturation in structural colors.^[^ ^37^, ^38^ ^]^ The same principle hinders the realization of high saturation artificial red structural color despite recent efforts to widen the gamut of structural colors.^[^ ^39^, ^40^, ^41^, ^42^, ^43^, ^44^, ^45^ ^]^ A class of high‐performance red color, called Schrödinger's red, requires zero reflection and perfect reflection correspondingly in different spectral ranges. A recent work^[^ ^41^ ^]^ uses double bound state in the continuum (BIC) modes to reduce the high‐order resonance modes in the blue/green region, resulting in high saturation red color in amorphous silicon (a‐Si) metasurfaces. However, due to the nature of BIC, which is based on the array effect, the red color can only be realized in a large array size and is highly polarization and viewing‐angle dependent, which would have severe limitations to some applications such as color display or printing. In this article, we show that the reflection induced by higher‐order modes can be eliminated by multipolar destructive interference in a properly designed free‐form resonant structure to achieve a red structural color with the highest saturation ever reported.

## 2 Results and Discussion

### 2.1 Overview of The Algorithm

To optimize the amorphous silicon (a‐Si) nanostructure with a red spectral response, we consider an n‐dimensional parameter space *S*, where *p* ∈ *S* is a design element defined by the geometrical parameters. Next, a DNN model is used to map the latent representation of these parameters. A figure of merit (FOM) function *f*[*r*(*p*, λ)] can be defined based on the optical response *r*(*p*, λ) of the structure, where λ is the wavelength. In this case, the targeted optical response is Schrödinger's red spectrum. The goal of the optimization algorithms is to find p* that satisfied p* =  *arg* *max*/*min* *f*[*r*(*p*, λ)]. For a generic photonics design problem, *f*[*r*(*p*, λ)] is typically inexplicit, and little assumption can be made about its properties. However, one could draw random samples from *f*[*r*(*p*, λ)] to explore its property. The evaluation of these samples is usually the bottleneck and computationally demanding. Therefore, it is essential to achieve the optimal design with as few samples as possible.

**Figure 1:**
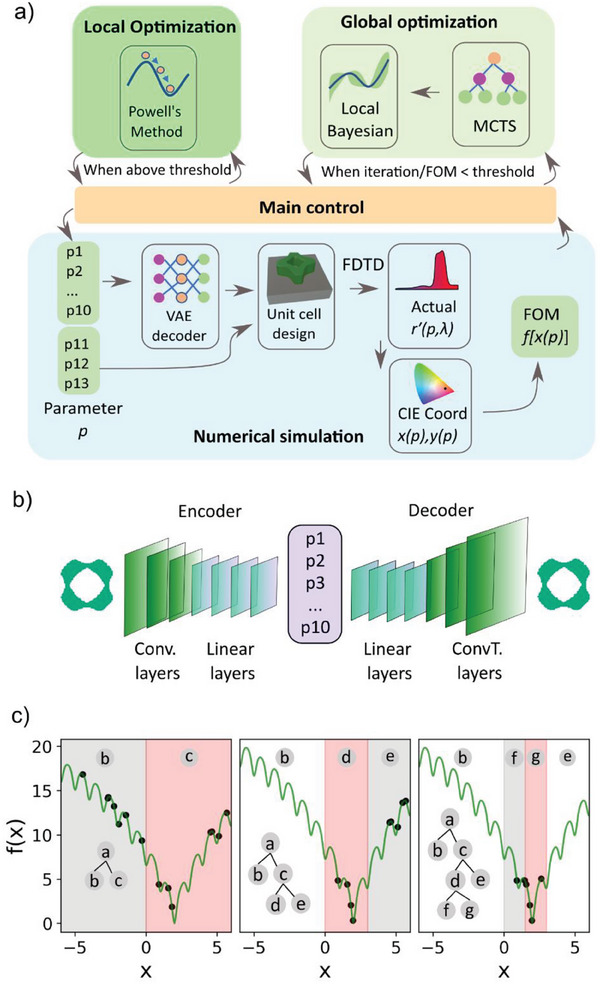
a) A schematic diagram of the proposed algorithm. b) Architecture of the variational autoencoder (VAE). c) Process for partitioning the search space using the global search algorithm. The pink‐shaded area represents the area with high FOM.

**Figure** **1**(a) illustrates the design model proposed in this work. The algorithm can be divided into four modules: the main control module, the numerical simulation module, the global optimization module, and the local optimization module. The main control module handles the whole optimization process, such as communicating with other modules and recording the optimization history. The numerical simulation module takes a vector as input and produces the FOM value. The local and global optimization modules suggest the next point to probe based on the current samples available.

### 2.2 Numerical Simulation Module

The input of the numerical simulation module is a 13D array *p*  =  [*p*_1_, *p*_2_, *p*_3_… *p*_13_]. Among them *p*_1_ −  *p*_10_ are latent parameters representing the 2D shape of the unit cell, and the rest of the parameters are the unit cell size, the nanostructure thickness, and the in‐plane scaling factor of the unit cell, respectively. The 10D latent parameters are mapped to a pixelated, binary‐valued image by the decoder network of a variational autoencoder (VAE), whose architecture is shown in Figure 1(b). The VAE consists of an encoder network, a bottleneck with 10 nodes, and a decoder network. The encoder consists of a series of convolutional (Conv.) layers and linear layers, while the decoder consists of transposed convolutional (ConvT.)  layers and linear layers. We generate random unit cell designs as the training data, and the network is trained to reconstruct these designs. As the data flows through the 10D bottleneck layer, the VAE learns an efficient compression of the training data, and subsequently, the bottleneck layer can be used as a latent representation of the design shapes. The detailed parameters of the VAE can be found in Section S1, (Supporting Information). An advantage of using VAE decoder is that the variation of the input parameters Δ*x* results in minute variations of the geometrical shape Δ*d*, which in turn results in a quasi‐continuous variation of the optical response *r*(*p*, λ).^[^ ^46^ ^]^ This is because DNNs are combinations of affine transformations and rectified linear units (ReLU), hence can be represented by piecewise smooth tropical geometries.^[^ ^47^ ^]^ This property is the foundation for our subsequent optimization process. The VAE decoder produces a wide variety of shapes with different topologies, as illustrated in Movie S1 (Supporting Information). Furthermore, the fabrication constraint, such as minimum feature size (i.e., 10 nm in this case) can be addressed by the VAE decoder during the data compression process. The level of details present in the structure can be adjusted by choosing the dimensionality of the latent representation layer.

Using FDTD, we obtain the broadband reflection spectra of the structure *r* (*p*, λ) = [*r*_1_, *r*,  *r*_3_,…,   *r*_100_] , where *r_i_* is the reflection at each discrete wavelength. The CIE tristimulus values X, Y, and Z, can be obtained by projection of the reflection spectrum onto each of the CIE 1931 two degree color match functions $\bar{x}$,$\bar{y}$,$\bar{z}$:^[^ ^48^, ^49^ ^]^

(1) $$\begin{array}{c} X\left(p\right)=\int r\left(p,\lambda \right)\bar{x}d\lambda ,\;Y\left(p\right)=\int r\left(p,\lambda \right)\bar{y}d\lambda ,\;Z\left(p\right)=\int r\left(p,\lambda \right)\bar{z}d\lambda \end{array}$$

Then the corresponding (*x*, *y*) coordinate in the CIE 1931 chromaticity diagram can be obtained by:

(2) $$x\left(p\right)=\frac{X\left(p\right)}{X\left(p\right)+Y\left(p\right)+Z\left(p\right)},\;y\left(p\right)=\frac{Y\left(p\right)}{X\left(p\right)+Y\left(p\right)+Z\left(p\right)}$$

Considering the color space has a triangular shape in the red region, one simply needs to maximize the *x* value to obtain the maximum red saturation: *f*[*x*(*p*)]  =  *x*(*p*).

### 2.3 The Global Optimization Module

The backbone of the global optimization module is the Monte Carlo tree search (MCTS), which has been used in other large‐scale artificial intelligence projects such as AlphaGo.^[^ ^50^ ^]^ We implemented La‐MCST^[^ ^51^ ^]^ to bifurcate the whole design space into good regions and bad regions so the search can be focused on the good regions. The process is shown in Figure. 1(c). Initially, some random samples [*p*,  *f*(*p*)] are drawn and represented by the root node *a*. The MCTS takes in all the samples and finds the best partition of the samples so that the selected samples have the highest average FOM : $\frac{1}{n}\sum_{i\;=\;1}^{n}f\left(p\right)$. The expansion of the root node into the child nodes *b* and *c* is accomplished by dividing the samples into two clusters by the k‐means clustering algorithm. In each expansion, the subset with a higher average FOM is given higher scores. One hundred iterations of rollouts are carried out to find the strategy of partitioning. The tree is expanded repeatedly until the number of samples in the node reaches a certain limit. A decision boundary is determined by support vector machines (SVMs) between the good and bad regions after the partition is complete. Subsequently, Bayesian optimization (BO) is used to determine the next sample to evaluate. This is achieved by drawing samples exclusively from the selected region using rejection sampling. Here SVMs are used for boundary determination because they converge faster and require fewer data in high dimensional to distinguish between good and bad regions. As the search tree grows, the search area is scaled down, and the estimation of the target landscape becomes more precise, and thus higher performance BO is achieved. The detailed flow chart for the global optimization module can be found in Section S2, (Supporting Information), and the source code is presented in Supplementary Information 3. With the suggested sample, the global control module calls the numerical simulation module to evaluate the suggested sample and starts the next iteration. After reaching a FOM threshold or iteration number, Powell's method^[^ ^52^ ^]^ is triggered as a local search algorithm to further optimize the FOM value. Here, we use Powell's method because it is a stable derivative‐free method and therefore, can be applied to a wide variety of photonics design problems.

### 2.4 Benchmarking of the Algorithm

The proposed algorithm is benchmarked against PSO, DE, and BO algorithms in two tasks: Schrödinger's red pixel design and the minimization of the Ackley function. For the first task, the bounds for *p*_1_ −  *p*_10_ is [−20, 20], and the bounds for *p*_11_ −  *p*_13_ are [0.2, 0.6], [0.08, 0.4], and [0.4,1] respectively. The scaling parameter, *p*_13_, are chosen to be smaller than unity to increase the spacing and thus to reduce the array effect. We use PSO, DE, and BO to call the numerical simulation module iteratively to maximize the FOM. The DE and PSO algorithm is taken from the open‐source code NEORL^[^ ^53^ ^]^ (https://neorl.readthedocs.io/en/latest/). The BO algorithm is from the open‐source code Bayesian Optimization^[^ ^54^ ^]^ (https://github.com/bayesian‐optimization/BayesianOptimization). The swarm size for PSO and DE is 20. For the PSO, we choose cognitive speed constant *c*1  =  2.05 and social speed constant *c*2  =  2.1. For DE, we chose mutation weight *F*  =  0.5, crossover probability *CR*  =  0.7. For the Bayesian optimization, the expected improvement acquisition function is used with ξ value of 0.05 to balance exploration and exploitation. The hyperparameters are chosen based on the suggestion of the respective codes to achieve the most stable optimization. The DE and PSO are run for 20 iterations, and the BO is run for 300 iterations.

**Figure 2:**
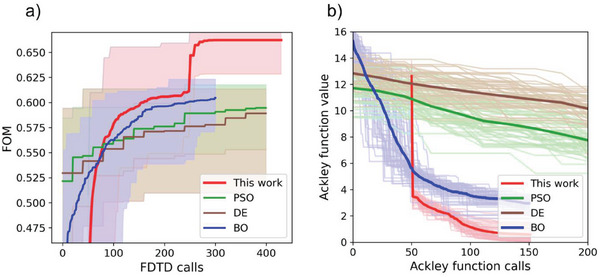
a – b) Comparison of the convergence speed between our algorithm and Particle Swarm Optimization (PSO), Differential Evolution (DE), and Bayesian optimization (BO) in Schrödinger's red pixel design task (a), and in solving the Ackley function (b). The red curve representing our algorithm is shifted by 50 to account for the initial samples. The light‐colored areas/lines represent the value for different iterations, while the thick‐colored lines are the averaged values.

Each algorithm is repeated 20 times, and the FOM values are shown in **Figure** **2**(a). The shaded area represents the maximum and minimum FOM, while the thick‐colored lines are the averaged FOM value. The result shows that our algorithm converges faster at around 300 FDTD calls and eventually reaches a higher FOM. The kink in the red curve represents the switch from global to local Powell's method, which indicates that local optimization is necessary to further improve the FOM. To further evaluate the performance of the proposed algorithm, it is benchmarked with the 13D Ackley function, which is a commonly used objective function to test optimization algorithms. The original Ackley function has a minimum value of 0 at [0, 0, 0…]. Considering many random generators generate random numbers with mean 0, we modify the Ackley function to have a minimum of 0 at [1,1,1…] to avoid bias caused by the random generators. The search boundary is [−10, 10] for all 13D, and the hyperparameters are the same as in the previous task. The code for the optimization of the Ackley function can be found in Supplementary Information 3. Each algorithm is run 50 times, and the target function values are plotted in Figure 2(b). The thick colored lines are the averaged function value. The target function reaches a lower value with fewer function calls using the proposed algorithm. Both PSO and DE maintain a dynamic population of samples, and all the samples are evaluated in each interaction. However, there are no strong mechanisms to ensure the next batch of samples is better than the previous one. This is the main source of inefficiency for these two algorithms. BO is considered an efficient tool in black‐box optimization when the target functions are expensive to evaluate.^[^ ^55^ ^]^ A surrogate model based on the Gaussian process is built for the target function, and the most promising samples are drawn based on the acquisition function. Our algorithm also uses BO to suggest the next sample to probe. However, in this case, the acquisition function is evaluated at a smaller, promising volume, thus improving the sampling efficiency.

### 2.5 Fabrication and Characterization of the Samples

**Figure 3:**
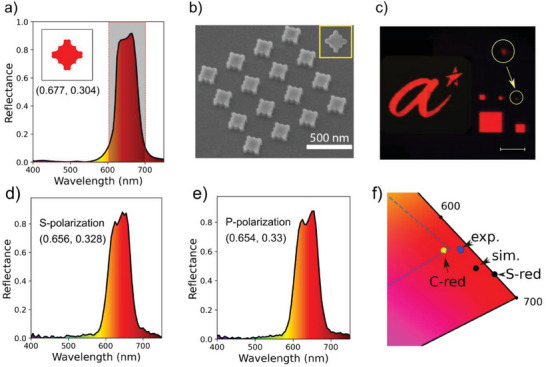
a) Optimized a‐Si nanostructure (inset) and its simulated reflection spectrum. The shaded area shows the spectrum of the ideal Schrödinger's red color. b) Tilted‐view (i.e., 30° along the vertical axis) scanning electron microscope (SEM) image of a fabricated a‐Si pixel. The sample was rotated 45° in the horizontal plane to show the side walls. The inset shows a top‐view SEM image of an a‐Si unit cell. c) Optical microscope image of the fabricated red pixels with various sizes (i.e., 30, 10, 5, 2.5, 1.5, and 0.4 µ*m* × µ*m*) and A*STAR logo made of the a‐Si nanostructures. The scale bar is 10 µ*m*. The inset is a magnified optical image of the 0.4 µ*m* pixel. d,e) Reflection spectra at normal incidence for s‐polarization and p‐polarization, respectively. f) The CIE coordinates of the optimized color in simulation (sim.) and experiments (exp.). The ideal Schrödinger's red pixel (S‐red) and Cadmium red (C‐red) are marked for reference. The dashed line is the boundary of the sRGB standard.

Using the optimization process described above, we derive an a‐Si nanostructure with a reflection spectrum that matches well with the ideal Schrödinger's red spectrum (i.e., our target optical response), as shown in **Figure** **3**(a). The optimal structure has a unit cell of 393 nm and a height of 120 nm. We then fabricate the a‐Si nanostructures using conventional e‐beam lithography and reactive‐ion etching processes. Figure 3(b) shows the scanning electron microscope (SEM) images of the fabricated a‐Si nanostructures. Detailed fabrication processes can be found in the Methods section. Figure 3(c) presents the optical microscope image of the fabricated structures showing high purity red color even for the smallest pixel made of 1 unit cell with a lateral dimension of ≈400 nm, corresponding to a printing resolution of ≈65000 pixels per inch. The simulated reflection spectra for different pixel sizes can be found in Section S1, Figure S4 (Supporting Information). The data show that the high‐purity color depends weakly on the pixel size and more on the scattering of the individual elements. Figure 3(d,e) shows the measured reflection spectra for the 30 µm pixel under s and p polarizations, respectively. It can be seen that, for both polarizations, the high reflection (i.e., > 85%) in the red region (i.e., 600 – 700 nm) and the suppression of the reflection in the shorter wavelength region (i.e., 400 – 600 nm) are achieved simultaneously, which agree well with the simulated reflection spectrum shown in Figure 3(a). The corresponding coordinates in the CIE 1931 color space are (0.656, 0.328) and (0.654, 0.33) for s and p polarization, respectively, which are slightly lower than the simulated structure (0.677, 0.304). This can be explained by the discrepancy in the designed and fabricated structures (Insets to Figure 3(a,b)). Nevertheless, these CIE coordinates are among the best red pixels ever reported.^[^ ^41^ ^]^ Furthermore, the color is polarization independent owing to the symmetry of the design. Figure 3(f) shows the color space coordinates of the a‐Si nanostructures in comparison with the ideal Schrödinger's red (S‐red) and Cadmium red (C‐red). It can be seen that the experimental value for a‐Si nanostructure in this work surpasses the pigment Cadmium red in terms of color purity, and the simulated value approaches that of the S‐red. With further improvement in nanofabrication to replicate the smaller features in the design, higher saturation of the red color can be achieved.

**Figure 4:**
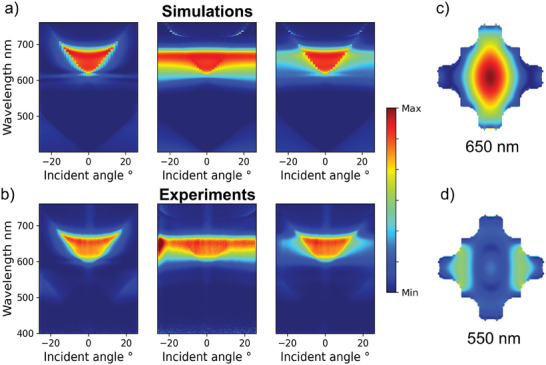
Simulated a) and measured b) angle‐resolved reflection spectra for 30‐µ*m* pixel. c,d) Normalized E‐field intensity distribution inside the a‐Si nanostructure at 650 and 550 nm, respectively.

The angle‐resolved reflection spectra are measured to investigate the performance of the a‐Si red pixel at a wider field of view, as shown in **Figure** **4**(a,b) for simulation and experiment, respectively. For p‐polarization, the red reflection band remains up to ± 18° viewing angle, while for s‐polarization, the high reflection band persists up to ± 26°. Remarkably, the suppression of reflection at shorter wavelengths (i.e., 400–600 nm) can be maintained over the whole measurement angle range (i.e., ± 26°). This can be attributed to the generalized multipolar interaction, which ensures the cancellation of outgoing waves at wider angles. The case for unpolarized light can be viewed as the superposition of p‐and s‐polarized light. The narrower viewing angle for p‐polarization can be explained by the lower efficiency to excite the horizontal electric dipole at higher incident angles. The normalized E‐field distributions within the structure at 650 nm (i.e., red region) and 550 nm (i.e., green region) are shown in Figure 4(c,d), respectively. It can be clearly seen that the field is much stronger for the case of 650 nm compared to that of 550 nm, indicating the suppression of resonance at lower wavelength region.

### 2.6 Multipolar Interactions

To shed more light on the optical responses of the a‐Si color structure, we studied the multipolar contributions to the scattering based on the induced electric current density. This can be expressed as *j* (***r***) =   − *i*ωε_0_(*n* ^2^ − 1) ***E*** (***r***), where ω is the angular frequency, ε_0_ is the vacuum permittivity, and ***E*** (***r***) is the electric field distribution within the unit cell. The induced current density is expanded into electric dipole (ED) ***p***, magnetic dipole (MD) ***m***, electric quadrupole (EQ) ***Q***, magnetic quadrupole (MQ) ***M*** and electrical octupole (EO) ***O*** in cartesian coordinates using the exact multipole expansion formulation.^[^ ^56^, ^57^, ^58^ ^]^ The reflection coefficient for x polarized incident plane wave can be obtained from the multipole moments using the following equation:^[^ ^57^ ^]^

(3) $$r=\frac{ik_{d}}{2E_{0}S_{L}\varepsilon_{0}\varepsilon_{d}}\;\left(p_{x}-\frac{1}{v_{d}}m_{y}+\frac{ik_{d}}{6}Q_{xz}-\frac{ik_{d}}{2v_{d}}M_{yz}-\frac{k_{d}^{2}}{6}O_{xzz}\right)$$

Here, $k_{d}=k_{0}\;\sqrt{\varepsilon_{d}}$ is the wave number in the surrounding medium, *E*_0_ is the incident electric fields, *S_L_* = *D* ^2^ , where *D* is the lattice constant for the square lattice. ε_0_ and ε_*d*_ are the vacuum and relative permittivity of the surrounding medium, respectively. The total reflection is calculated as *R*  = |*r*|^2^ . To visualize the multipole interaction, we factor in all coefficients and represent the reflection coefficient as follows:

(4) $$r=\left|C\right|\;\left(A_{p}e^{i\varphi_{p}}+A_{m}e^{i\varphi_{m}}+A_{Q_{e}}e^{i\varphi_{Qe}}+A_{Q_{M}}e^{i\varphi_{Qm}}+A_{O_{e}}e^{i\varphi_{Oe}}\right)$$

Where *A_i_* and φ_*i*_ (with *i*  =  *p*, *m*, *Q_e_*,*Q_m_*, *O_e_*) are the magnitude and phase of each multipole component, respectively.

**Figure 5:**
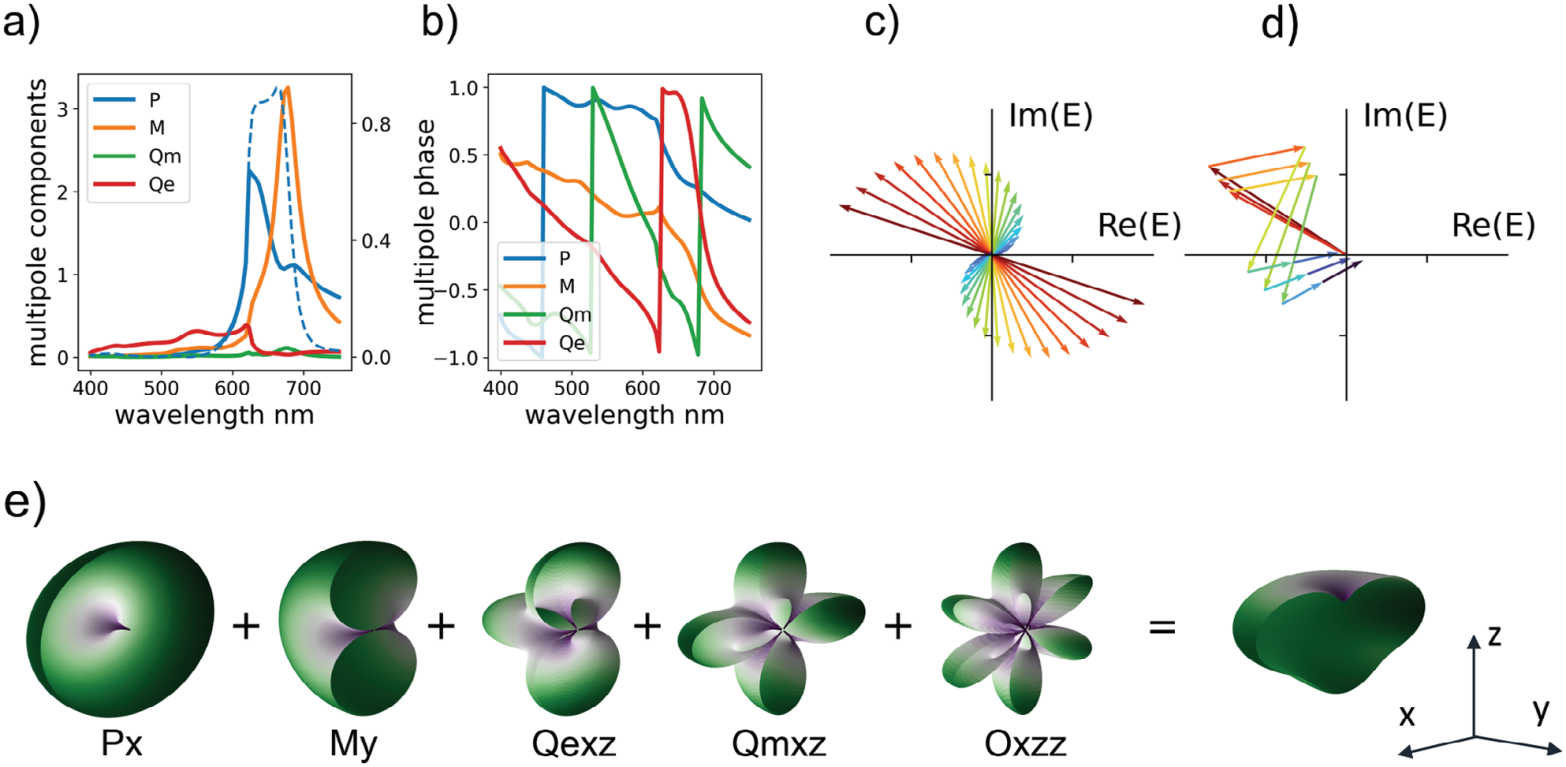
a) Multipolar decomposition for the optimized reflection spectra in a‐Si nanostructure; The solid lines are multipole magnitude, and the dashed line is the reflection spectra. b) The phase spectra of the multipoles in (a). c) The cancellation condition for the case of two‐multipole interference. d) The cancellation condition for multiple components interference at three different wavelengths: 548, 549, and 550 nm. Each arrow represents one multipolar component. e) Far‐field distribution of the multipolar components and their interference pattern.

As shown in **Figure** **5**(a,b), the electric dipole and magnetic dipole responses dominate the high reflection band in the red region (i.e., >600 nm), while the EQ mode is dominant at the shorter wavelength region (i.e., <600 nm). In the optimized structure, the magnitude of EQ response is low and is further suppressed by the interaction with other multipolar components. Figure 5(c) shows the destructive interference conditions for the case of two multipoles, which generally require opposite phases and the same magnitude. Since each multipolar component has different wavelength dependence, it is challenging to achieve broadband cancellation for two multipoles. However, it is possible in the case of multiple multipole interactions. Figure 5(d) shows that the cancellation condition for three different wavelengths can be achieved by varying the phase and amplitude of each multipole component. Furthermore, the cancellation also guarantees high suppression of the reflected field at a wide angle, as shown in the far field distribution in Figure 5(e). We further discuss the tuning power of the multipoles by comparing the optimized structure with various sub‐optimized ones in Section S6 and Figures S5–S7 (Supporting Information). The result shows that the small variation of the geometries can effectively tune the multipole moments and phases to reach the cancellation condition at a shorter wavelength and enhancement condition at a longer wavelength. Controlling of two multipoles components is critical in many fascinating phenomena such as Fano resonances,^[^ ^59^ ^]^ electromagnetically‐induced‐transparency,^[^ ^60^ ^]^ unidirectional scattering,^[^ ^61^, ^62^ ^]^ Janus and Huygens dipoles,^[^ ^63^ ^]^ and perfect absorption,^[^ ^64^ ^]^ to name just a few. They originate from the different parity and polarization signatures of each multipole radiation pattern. However, previous reports rely on two multipole components with a specific phase and amplitude relationship at a specific wavelength. The freeform structures allow for more general interaction involving multiple multipoles with generic phase and intensity relationships, providing a possibility for other wide bandwidth photonic devices.

## 3 Conclusion

We designed and experimentally demonstrated a‐Si structural red color with a record‐high performance by employing an efficient data‐driven machine learning approach. The optimal free‐form structure enables the elimination of high‐order resonances in the a‐Si structure due to their destructive interferences, leading to the highest ever reported saturation of red color with a CIE coordinate of (0.677, 0.304) in simulation and ≈(0.656, 0.328) in experiments. Remarkably, the color structures have low polarization dependence and high viewing angle (i.e., up to 26°), which are unprecedented for such high saturation color.^[^ ^41^ ^]^ The introduction of a simple bifurcation rule and the use of SVMs in determining good and bad optimization regions are the keys to rapid convergence. The design model proposed in this work significantly advances the free‐form design approach for high‐performance resonant nanostructures, which may find broad applications in color filters, spectrometers, and sensors.

## 4 Experimental Section

### Numerical Simulation

Commercial software Lumerical FDTD was used to obtain the reflection spectrum of the Si structures by assuming periodic boundary conditions. The machine learning algorithm was implemented in Python using the Pytorch open package. The refractive index of a‐Si was based on the ellipsometry measurements.^[^ ^65^ ^]^ The details for multipolar expansion can be found inSection S4 (Supporting Information).

### Nanofabrication of Si Color Pixels

Amorphous silicon film was grown onto a thin quartz substrate using chemical vapor deposition (CVD, OPIT Plasmalab 380). The thickness of the deposited film was then characterized using a reflectometer (Filmetrics F20). The measured thickness of a‐Si film was ≈123 nm, which was close to the designed value of 120 nm. The sample was then patterned using a standard electron‐beam lithography (EBL) process: First, hydrogen silsesquioxane (HSQ) resist (Dow Corning, XR1541‐002) was spin‐coated on the a‐Si/quartz substrate at 3000 RPM, giving a ≈150 nm‐thick film. A charge dissipation layer (Espacer 300AX01) was used to minimize the charging effect. The nanopatterning was done using the Elionix ELS‐7000 system at 100 kV and 500 pA. The EBL pattern was then developed using a home‐made salty developer.^[^ ^66^ ^]^ Finally, the nanopatterns were transferred to the a‐Si layer by a reactive‐ion‐etching process (OPIT Plasmalab 100) using HBr and O_2_ gases.

### Optical Characterizations

Angle‐resolved reflection measurements of the Si color pixels were conducted using a home‐built back‐focal plane setup consisting of an inverted microscopy (Nikon Ti‐U) and a spectrograph (Andor SR‐303i) coupled with an Electron Multiplying Charge‐Coupled Detector (EMCCD) (Andor Newton 971, 400×1600 pixels). A detailed schematic of the setup was described in the previous work.^[^ ^67^ ^]^ A 50× objective (Nikon) with a numerical aperture (NA) of 0.45, corresponding to a viewing angle of ± 26.7°, was used for illumination and collection of the reflection spectra. A pinhole was inserted into the optical path at an image plane to limit the collection area before projecting to the spectrometer slit. Reflected light was normalized to light reflected from a silver mirror in the same measuring configuration after accounting for photodetector noise effects (dark current subtraction).

### Statistical Analysis

To compare the proposed algorithm with BO, PSO and DE. The same problem was optimized with each method 20–50 times until the algorithms converge. The FOM values were recorded each time the algorithms call the FDTD function. The FOM values during the whole optimization process were plotted in Figure 2(a,b) without any normalization or processing. Thus, the maximum and minimum values of the FOM were used as an indication of the statistical performance of each algorithm.

## Conflict of Interest

The authors declare no conflict of interest.

## Supporting information

Supporting Information

Supplementary Movie S1

## Data Availability

The data that support the findings of this study are available from the corresponding author upon reasonable request.
