# Supplementary material for: Schrödinger's Red Beyond 65,000 Pixel‐Per‐Inch by Multipolar Interaction in Freeform Meta‐Atom through Efficient Neural Optimizer
Source: Adv Sci (Weinh). 2023 Dec 13;11(13):2303929. doi: 10.1002/advs.202303929 (PMC10987134; doi:10.1002/advs.202303929)
Supplement: Supplementary file 1 — Supporting Information [file ADVS-11-2303929-s002.pdf]

ADVANCED SCIENCE
Open Access

Supporting Information

for *Adv. Sci.*, DOI 10.1002/advs.202303929

Schrödinger's Red Beyond 65,000 Pixel-Per-Inch by Multipolar Interaction in Freeform Meta-Atom through Efficient Neural Optimizer

Ronghui Lin, Vytautas Valuckas, Thi Thu Ha Do, Arash Nemati, Arseniy I. Kuznetsov, Jinghua Teng and Son Tung Ha**

Supplementary Information 1

Schrödinger's red beyond 65,000 pixel-per-inch by multipolar interaction in freeform meta-atom through efficient neural optimizer

Ronghui Lin†, Vytautas Valuckas†, Thi Thu Ha Do, Arash Nemati, Arseniy I. Kuznetsov, Jinghua Teng, Son Tung Ha**

This PDF file includes:

Supplementary Text, section 1- 6
Figs. S1 to S7

Other Supplementary Materials for this manuscript include the following:

- Supplementary Information 2: Movies S1 showing the VAE decoder production of various geometry of the unit cell.
- Supplementary Information 3: Source code of the global optimization module: (https://github.com/esperancelin/Global-optimization-of-Ackley-function.git)

Supplementary Text

1. Parameters of the VAE

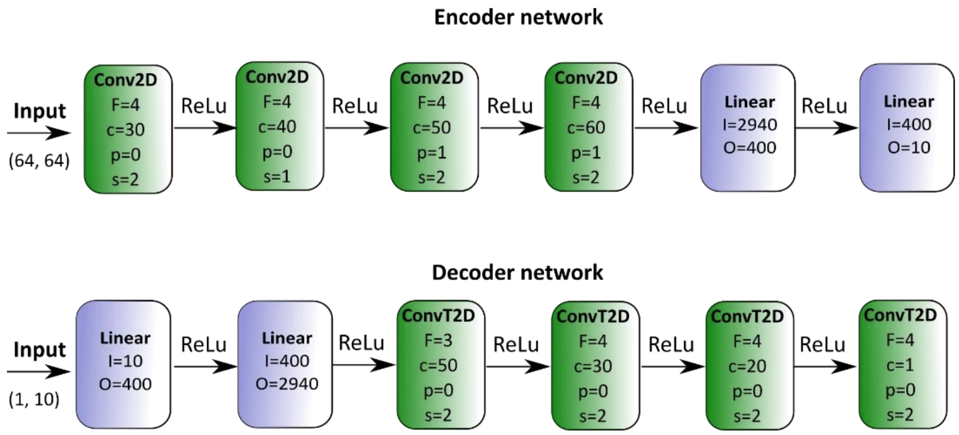

Figure. S1. The architecture of the VAE

The detailed parameter of the VAE network is shown in Figure S1. The input of the encoder is a 64 by 64 binary image of 0 or 1 representing the unit cell design. The encoder consists of 4 layers of 2D convolutional (conv.) layers and 2 layers of linear layers. The filter size, the number of channels, the padding size, and the stride are represented by "F", "c", "p", "s" respectively. The letter "I" and "O" represents the input and output size of the linear layer. The input of the decoder is a 10-dimensional array. The input is passed through two linear layers and 4 transposed convolutional layers to reconstruct the original image with 64 by 64 pixels. During the training, We constructed a geometrical library with 20000 unit cell designs as the

training data. The VAE can pick up the pattern and produce unit cell designs with the same C4v symmetry. After training, the decoder can be used to map array inputs into geometrical shapes.

2. The global optimization module

Here we give a detailed description of the global optimization module of the proposed algorithm. As shown in Figure S2, The global optimization module consists of two submodules, namely the MCTS module and the local Bayesian module. The MCTS takes in all the samples taken so far and runs a search algorithm to find the best partition of the samples so that the selected samples have the highest average FOM : $\frac{1}{n}\sum_{i=1}^{n} f(p)$. The selected samples represent the most promising area to search. After the partition, a local Bayesian process provides the next sample to evaluate within the promising area.

The MCTS follows the standard procedure: selection, expansion, rollout, and backpropagation. In each iteration, the MCTS selects a node based on the upper confidence bound (UCB), which is a standard way of choosing the best node. In the expansion process, each node is split into a predefined number of children nodes by using the K-means clustering algorithm implemented by the open-source scikit-learn library [1,2]. Each node represents a different way of splitting the existing samples. During the rollout, the splitting algorithm is called progressively until the number of samples in a node reaches a certain limit.

The average FOM of the leaf nodes $\frac{1}{n}\sum_{i=1}^{n} f(p)$ are backpropagated to the selected node to select the best partition strategy. Since all the data points are not clustered in high dimensions, different random states are used to get different divisions. The number of samples is used as a threshold to determine whether a node is splittable. It also controls the ratio between exploration and exploitation. If the threshold is big, the search area is also big. In contrast, if this number is small, the area is contracted to a smaller area. After the MCTS picks out a set of samples. An SVM can be trained to determine the boundary between the selected sample and the rest of the sample. Bayesian optimization process is used subsequently to suggest a new sample for the next evaluation in the promising area. We first train a Gaussian process regressor with the full set of samples to build up a surrogate model, after which we maximize the acquisition function to give the next sample to evaluate. To sample for the acquisition function, the pre-trained SVM is used as a reference for rejection samples to get samples distributed in the picked volume. In this way, the BO is confined to a promising region and thus improves the optimization efficiency. The acquisition function is either expected improvement or probability of improvement acquisition function. The main control takes the suggested sample and calls the numerical simulation module to evaluate the FOM. Afterwards, the FOM and the sample are added to the existing sample pool and a new iteration of optimization is started. This global optimization iterates until the FOM or the number of iterations reaches a threshold value.

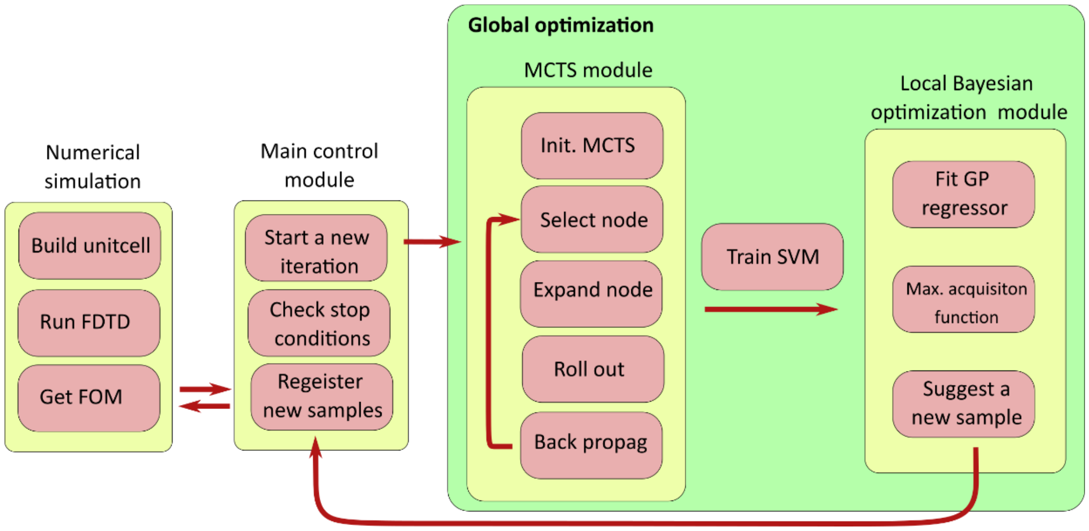

Figure. S2. The flowchart for the global optimization module

3. Performance bechmarking with other works

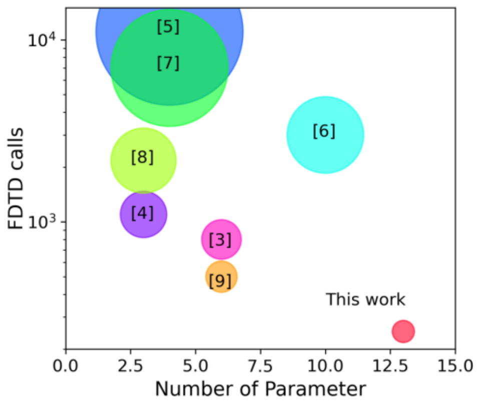

Figure. S3. Comparison of the algorithm efficiency with prior works using RL.

Figure. S3 compares our algorithm with previous reports [3–9] using RL and heuristics optimization algorithms. The typical number of function calls are in the 10^3 range with a much smaller design parameter size for previous reports, as opposed to around 300 function call in 13-dimensional space in this work. The high efficiency in data usage and convergence speed is attributed to the combination of the efficient global algorithm and the local optimization algorithm.

4. Multipole expansion

The multipole expansion is carried out in Cartesian coordinate based on the displacement currents $\mathbf{j} = \mathrm{i}\omega\varepsilon_0(\varepsilon_r - 1)\boldsymbol{E}$, where ω is the angular frequency, ε_0 is the permittivity in vacuum, and ε_r is the complex relative permittivity. The multipole moments are calculated as follows:

Electric dipole moment: $\mathbf{P} = \frac{1}{i\omega}\int \boldsymbol{j}\, d^3r$

Magnetic dipole moment: $\mathbf{M} = \frac{1}{2c}\int (\boldsymbol{r} \times \boldsymbol{j}) d^3r$

Toroidal dipole moment: $\boldsymbol{T} = \frac{1}{10c}\int[(\boldsymbol{r}\cdot\boldsymbol{j})\boldsymbol{r} - 2r^2\boldsymbol{j}]d^3r$

Electric quadrupole moment: $Q_{\alpha\beta} = \frac{1}{i\omega}\int[r_\alpha j_\beta + r_\beta j_\alpha - \frac{2}{3}\delta_{\alpha\beta}(\boldsymbol{r}\cdot\boldsymbol{j})]\,d^3r$

Magnetic quadrupole moment: $\mathrm{M}_{\alpha\beta} = \frac{1}{3c}\int[(\boldsymbol{r}\times\boldsymbol{j})_\alpha r_\beta + (\boldsymbol{r}\times\boldsymbol{j})_\beta r_\alpha]\,d^3r$

The total radiation power of each multipole moment is:

$$\mathrm{I} = \frac{2\omega^4}{3c^3}|\boldsymbol{P}|^2 + \frac{2\omega^4}{3c^3}|\boldsymbol{M}|^2 + \frac{4\omega^5}{3c^4}|\boldsymbol{P}\cdot\boldsymbol{T}|^2 + \frac{2\omega^6}{3c^5}|\boldsymbol{T}|^2 + \frac{\omega^6}{5c^5}\sum\left|Q^e_{\alpha\beta}\right|^2 + \frac{\omega^6}{40c^5}\sum\left|Q^m_{\alpha\beta}\right|^2 + o(\frac{1}{c^5})$$

Here, c is the speed of light. α, β, γ = x, y, z are the Cartesian coordinates.

5. <u>Size dependent reflection</u>

Figure S4(a) depicts the reflection spectra for different array size. The suppression of reflection at wavelength below 600 nm is consistent for all the array size. The reflection peak in the red band due to the response is also present for the smallest array simulated. The dropped reflection when the array gets smaller is due to the limitation of the monitor size during the simulation, which can only capture part of the reflected light. As the array size get smaller, the scattered field resembles more spherical wave, and only a small portion of the scattered field can be captured by the field monitor. However, it is clear from the measured optical microscope data, even a single unit cell shows high saturation red color. Figure S4(b) shows the corresponding CIE colors for various array size. As the array size gets bigger, the coordinates move towards the red region. It is estimated that a color saturation comparable to Cadmium red can be obtained with an array size of 6 by 6-unit cells. Figure S4(c-d) shows the multipole decomposition of the scattering cross section of a single scatterer. The dashed line in Figure S4(c) is the scattering cross section, it shows a pronounced peak at 600-700 nm range, which coincides with the enhancement of reflection in the array structures. Moreover, we also observe the cancellation of the quadrupole components in the lower wavelength range, similar to that of the array. This indicates that the single unit cell is able to reach certain cancellation and the array effect is less important in our structure.

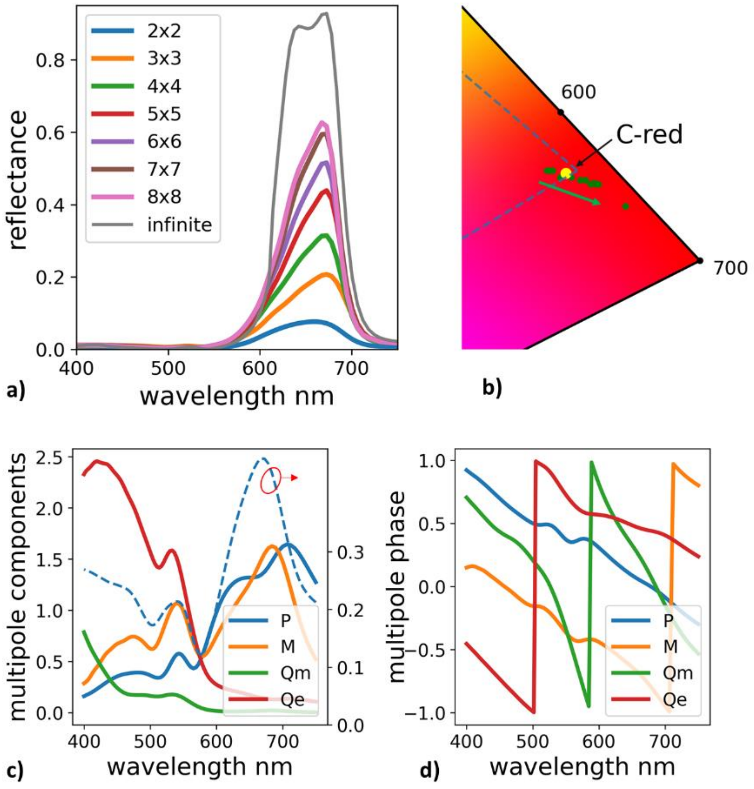

Figure. S4. (a) The simulated reflection spectra for different array size. (b) The green dots show the CIE coordinates for the array of different size. The Cadmium red (C-red), are also shown for reference. The dashed line is the boundary of sRGB standard. And the green arrow indicates the increasing array size. (c) The multipole components and the scattering cross section of a single isolated unit cell. (d) The multipole phase of the scattering cross section of a single isolated cell.

6. Comparison of the optimized structure with sub-optimized structures

In this section, we show the comparison of the optimized structure with various unoptimized structure to illustrate the role of the multipolar cancellation. In all the structures shown in Figure S5, the volume is conserved while only the arrangement of the material is altered. In all these structures, the reflection peak at the red region remains high. However, there is substantial reflection in the shorter wavelength region for the unoptimized structures, as shown in Figure S5(a, b). The reflection at shorter wavelength results in the CIE coordinate moving toward the blue and yellow region and therefore lower red saturation. The effective cancellation of the reflection at lower wavelength is due to the minute variation of the multipole moments and multipole phase as shown in Figure S5, last two columns to the right.

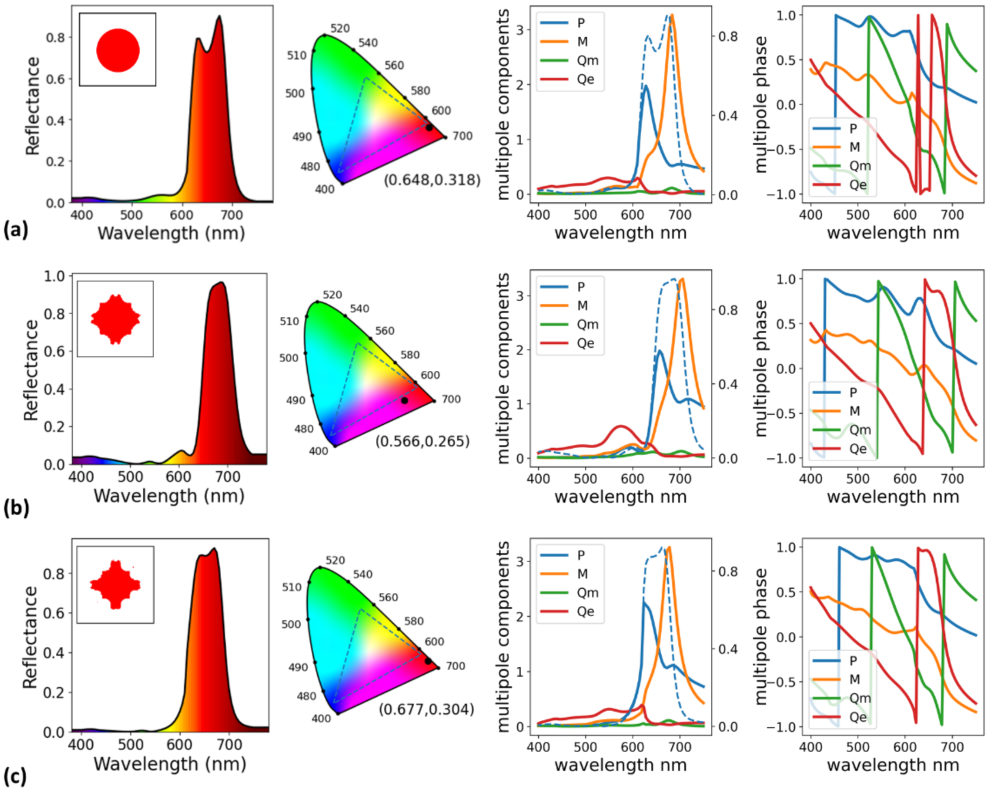

Figure. S5. Comparison of different geometries. (a) Circular geometry with the same volume as the optimized geometry. (b) Complex geometry with slight variation. (c) The optimized geometry. The rows in each panel are simulated reflection spectra, CIE color coordinate, multipole moments and multipole phase respectively.

The electric field intensity distribution is very similar at longer wavelength (680 nm) for the optimized structures and a slightly altered one (Figure S6). As the wavelength get shorter, the difference become more significant, which is also consistent with the previous observation. This further confirms the variation of the geometries can effectively modulate the optical response in a broadband wavelength.

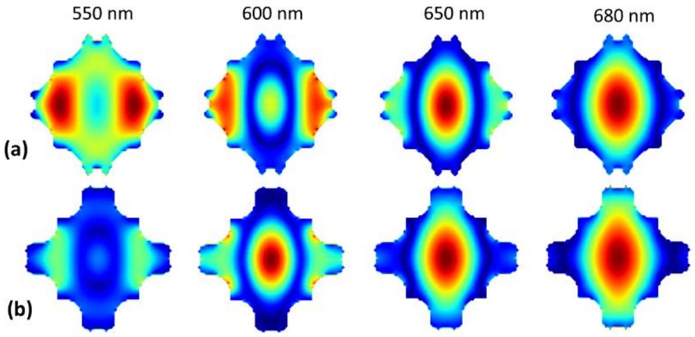

Figure. S6. Comparison of the E field distribution at different wavelength between (a) sub-optimized structure and (b) optimized structure.

Figure S7 (a, d) shows the comparison of the SEM images of the optimized structure and the sub-optimized one, respectively. The differences in the geometry are subtle, but the optical response is quite different, especially at shorter wavelength. Figure S7(e) shows the measured

reflection spectrum of the sub-optimized structure. It can be seen the reflection in the shorter wavelength is much higher than the optimized structure, resulting in a CIE coordinate of (0.645,0.326) which is worse than the optimized one as shown in Figure S7 (c, f).

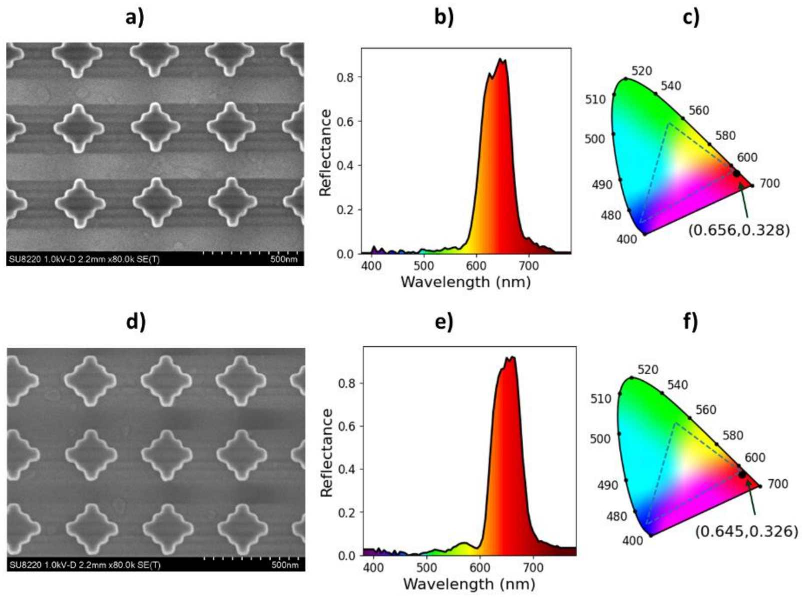

Figure. S7. SEM image, reflection spectrum, and CIE coordinate of the optimized structure (a-c) and the sub-optimized structure (d-f), respectively.

References

[1] L. Buitinck, G. Louppe, M. Blondel, F. Pedregosa, A. Mueller, O. Grisel, V. Niculae, P. Prettenhofer, A. Gramfort, J. Grobler, *ArXiv Prepr. ArXiv13090238* **2013**.

[2] Fabian Pedregosa, Gaël Varoquaux, Alexandre Gramfort, Vincent Michel, Bertrand Thirion, Olivier Grisel, Mathieu Blondel, Peter Prettenhofer, Ron Weiss, Vincent Dubourg, Jake Vanderplas, Alexandre Passos, David Cournapeau, Matthieu Brucher, Matthieu Perrot, Édouard Duchesnay, *J. Mach. Learn. Res.* **2011**, *12*, 2825.

[3] C. Luo, S. Ning, Z. Liu, Z. Zhuang, *Extreme Mech. Lett.* **2020**, *36*, 100651.

[4] J. Park, S. Kim, J. Lee, S. G. Menabde, M. S. Jang, *Phys. Rev. Appl.* **2019**, *12*, 024030.

[5] H. Liang, Q. Lin, X. Xie, Q. Sun, Y. Wang, L. Zhou, L. Liu, X. Yu, J. Zhou, T. F. Krauss, J. Li, *Nano Lett.* **2018**, *18*, 4460.

[6] J. Li, L. Bao, S. Jiang, Q. Guo, D. Xu, B. Xiong, G. Zhang, F. Yi, *Opt. Express* **2019**, *27*, 8375.

[7] W.-K. Lee, S. Yu, C. J. Engel, T. Reese, D. Rhee, W. Chen, T. W. Odom, *Proc. Natl. Acad. Sci.* **2017**, *114*, 8734.

[8] I. Sajedian, T. Badloe, J. Rho, *Opt. Express* **2019**, *27*, 5874.

[9] I. Sajedian, H. Lee, J. Rho, *Sci. Rep.* **2019**, *9*, 10899.
