# Supplementary figures and images for: Schrödinger's Red Beyond 65,000 Pixel‐Per‐Inch by Multipolar Interaction in Freeform Meta‐Atom through Efficient Neural Optimizer
Source: Adv Sci (Weinh). 2023 Dec 13;11(13):2303929. doi: 10.1002/advs.202303929 (PMC10987134; doi:10.1002/advs.202303929)

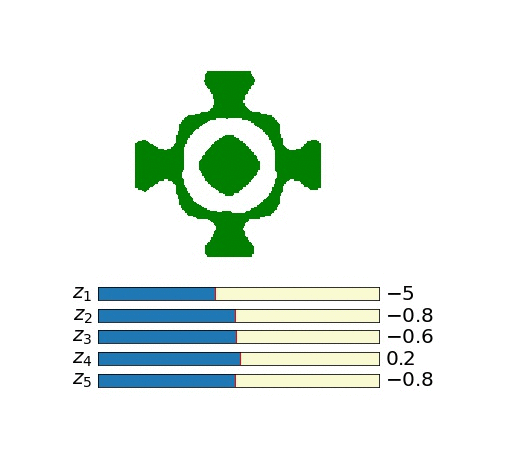

Supplement: Supplementary file 2 — Supplementary Movie S1 [file ADVS-11-2303929-s001.gif]
